# Subglacial discharge plume behaviour revealed by CTD-instrumented ringed seals

**DOI:** 10.1038/s41598-018-31875-8

**Published:** 2018-09-07

**Authors:** Alistair Everett, Jack Kohler, Arild Sundfjord, Kit M. Kovacs, Tomas Torsvik, Ankit Pramanik, Lars Boehme, Christian Lydersen

**Affiliations:** 1 Norwegian Polar Institute, Fram Centre, N-9296 Tromsø, Norway; 2 ESSO-National Centre for Antarctic and Ocean Research, Headland Sada, Vasco da Gama, Goa 403804 India; 3 NERC Sea Mammal Research Unit, Scottish Oceans Institute, University of St Andrews, St Andrews, UK

## Abstract

Subglacial discharge plumes increase submarine melting of marine-terminating glaciers significantly; however, *in-situ* data on their properties and behaviour are limited. We present oceanographic data collected by ringed seals (*Pusa hispida*) instrumented with GPS-equipped conductivity-temperature-depth satellite relay data loggers (GPS-CTD-SRDLs) in Kongsfjorden, Svalbard, during 2012. The seals foraged just outside the plumes and collected hydrographic data from within the plumes’ upwelling cores as they returned to the surface. The seals encountered water with fractions of subglacial discharge as high as 27% at 60 m below the ocean surface. The ringed seals responded rapidly to spatial and temporal variations in subglacial discharge at the glacier terminus, suggesting that prey becomes available quickly following the appearance of plumes. The seals’ dive locations were used to monitor the presence of plumes over a four-month period. High surface runoff from Kronebreen catchment created strong plumes, but weak plumes were present even during periods of low surface runoff. The continued retreat of Kronebreen, and other tidewater glaciers, will lead to the loss of these marine-termini as the glaciers retreat onto land. The techniques presented here improve our understanding of the drivers of glacial retreat and the implications of future habitat loss for glacier-associated birds and mammals.

## Introduction

Subglacial discharge plumes are frequently observed at the fronts of marine-terminating glaciers. These plumes are located at the ice-ocean boundary, forming a link between the complex subglacial hydrological systems of tidewater glaciers and the fjords into which they drain. Plumes of subglacial discharge enhance submarine melting^1–3^, play a role in glacier calving and terminus morphology^4^, contribute to fjord circulation^5–8^, and have been used to infer the morphology of the subglacial hydrological system^9,10^. Plumes also play an important role in the ecosystems within glacial fjords. Seabirds, particularly kittiwakes (*Rissa tridactyla*) and fulmars (*Fulmarus glacialis*), focus their foraging in the so-called “brown-zone”, where sediment-laden water contained in plumes reaches the surface^11–13^. Marine mammals, such as white whales (*Delphinapterus leucas*) and ringed seals (*Pusa hispida*), also preferentially forage close to glacier fronts^14,15^, though no specific link to subglacial discharge plumes has yet been established. Despite the remaining uncertainties, it is clear that plumes play an important role in the glaciology, oceanography and biology of fjords with large marine-terminating glaciers, and the tendency of marine mammals to dive in these areas means that they can be used to collect valuable oceanographic data from their environments^16,17^.

Our understanding of subglacial discharge plumes is largely based on modelling^3,5,18,19^, surface observations^9,10^ and oceanographic data collected in horizontal outflow areas downstream of the upwelling plumes^20–22^. Recently, two attempts have been made to collect data from within the vertical upwelling regions of plumes^23,24^. However, in both of these studies the instruments used were rapidly pushed out of the plume core due to the rapid horizontal export of water at the surface. As a result, there is only a limited amount of field data available to incorporate into modelling efforts, and therefore plumes are difficult to include in existing large-scale models of either ice or fjord dynamics. A recent modelling study of Kongsfjorden^25^, which did not include a fully-developed plume representation, showed that the simulated fjord circulation is likely incorrectly represented in the peak runoff season. This was most noticeable in the vicinity of the glacier fronts, but may also have affected the exchanges with the open ocean, even after the subglacial discharges cease at the end of summer. The only current implementation of a plume in a fjord model relies on a parameterisation of plume dynamics based upon plume theory^6^. While this is a valuable tool for fjord-scale circulation studies, there are still many unknowns in how to parameterise the non-idealised conditions of plumes at glacier termini using plume theory. *In-situ* data from within plumes is therefore vital to constrain and improve existing parameterisations.

In this paper, we present data collected from ringed seals instrumented with GPS-equipped conductivity-temperature-depth satellite relay data loggers^26^ (GPS-CTD-SRDLs) that foraged close to glacier fronts in Kongsfjorden, Svalbard during 2012. We compare the spatial and temporal variations in the locations of the seal-collected profiles to surface runoff from the Kronebreen catchment and use a high-resolution plume model to interpret the oceanographic profiles. The data collected by the GPS-CTD-SRDLs provide a valuable and unique dataset from the upwelling region of subglacial discharge plumes as well as highlighting an important foraging ground for ringed seals.

## Study Area and Timing

**Figure 1:**
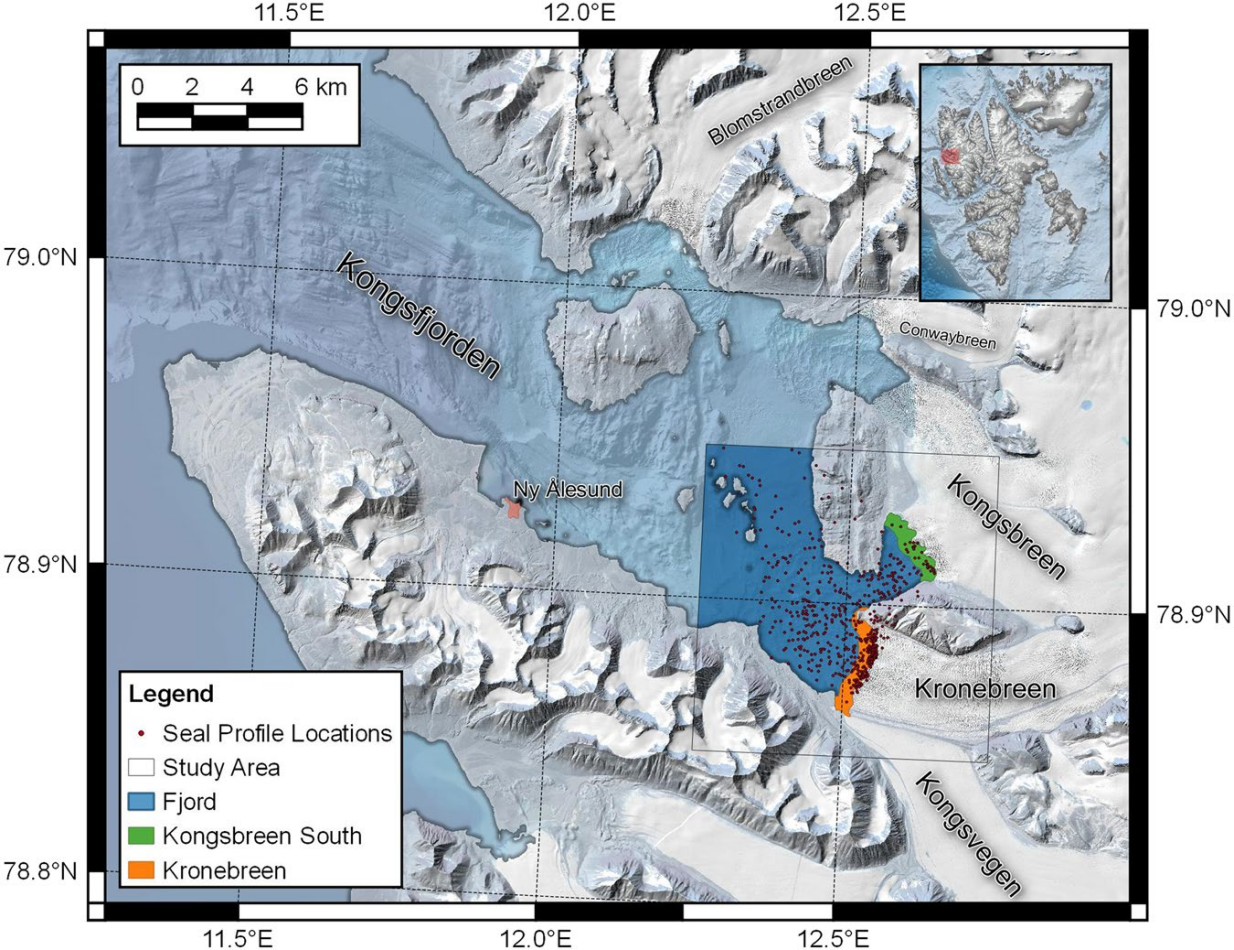
The study area in Kongsfjorden, Svalbard, showing the location of seal-collected CTD profiles. The fjord region (dark blue) and the area less than 500 m from the terminus of Kronebreen (orange) are identified with colours corresponding to those in Fig. 5. The region within 500 m of the terminus of Kongsbreen South is highlighted in green.

Our study focussed on the inner region of Kongsfjorden (Fig. 1), in western Svalbard. The study area contains two glacier termini: the Kronebreen-Kongsvegen terminus, which is currently dominated by Kronebreen, and the southern branch of Kongsbreen, which terminates in a small bay to the north of Kronebreen. Kronebreen is a marine-terminating glacier grounded in water around 100 m deep; the southern section of the terminus is much shallower than the northern section. Kronebreen responds strongly to oceanic forcing^27^ and is characterised by a number of plumes of subglacial discharge throughout the summer months^28–30^. Kongsbreen South, on the other hand, has retreated onto land but is still characterised by large sediment plumes that are now largely driven by surface inputs of glacial discharge.

The study covered the period between August and December 2012, and therefore captured the latter half of the melt season for the glaciers draining into Kongsfjorden. This is the period of minimum sea ice extent in the Arctic, and therefore the time when glacier fronts become most important to ringed seals and other ice-associated fauna. Additionally, the instruments were glued to the hair of the seals, and thus the timing of the study was partially dependent on the timing of the seals’ annual moult, which is normally completed in June or July.

## Results

### Spatial distribution of dive profiles

**Figure 2:**
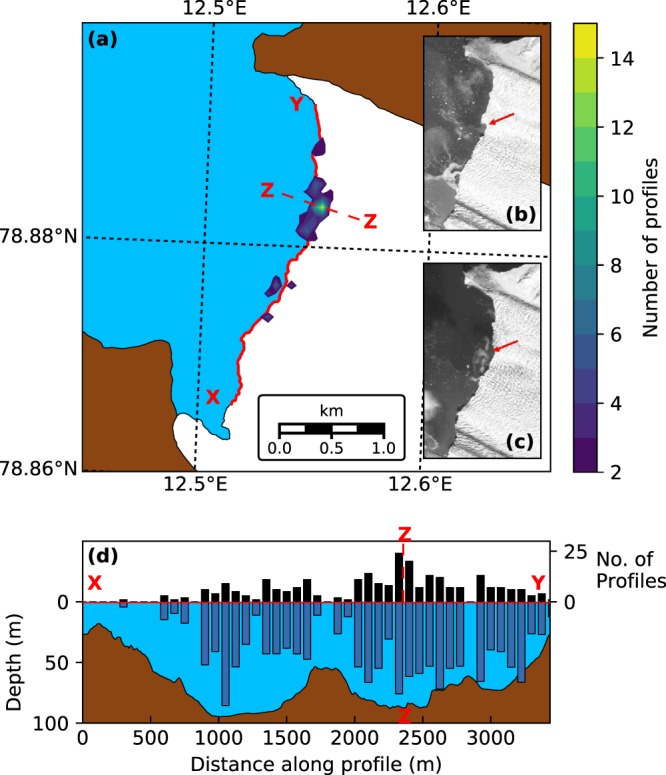
(**a**) Shaded contours of seal-collected profile density, calculated using 100 × 100 m bins, showing increased activity around plume locations at the terminus of Kronebreen. Inset are two FORMOSAT images showing plume location from (**b**) 11 July and (**c**) 24 August 2012. (**d**) Number of profiles collected within 500 m of the terminus (black bars) and mean dive depth (blue bars) in 100 m bins along section XY. Bathymetry along the profile is also shown. Line ZZ marks the peak in dive density along section XY.

Five ringed seals were instrumented with GPS-CTD-SRDLs and collected data within the study area between August and December 2012 (Supplementary Table S1). The data from these seals were included in a four-year study of ringed seals around Svalbard, which showed that the seals typically foraged within five km of glacier fronts and spent approximately 70% of their time in these regions^31^. The behaviour was consistent in all four years, showing that the data presented here from 2012 are representative of ringed seal behaviour across other years. More details on the capturing and handling techniques used to tag the seals can also be found in this previous study. Throughout the 2012 study period, which this work focusses on, at least four out of the five seals were present within the study area at all times. The profiles collected with the GPS-CTD-SRDLs were filtered to retain data which met certain quality criteria (see Methods); a total of 648 profiles within the study area met the criteria and were analysed further. Frontal positions for Kronebreen and Kongsbreen South were digitised from a two-metre resolution FORMOSAT image collected on 24 August 2012 (Fig. 2c), shortly after the beginning of the study period.

During the study period, 43% (277 out of 648) of the profiles were collected within 500 m of the terminus of Kronebreen, while 5% (32 out of 648) were collected within 500 m of Kongsbreen South. The remaining 52% (339 out of 648) of dives were collected more than 500 m from the glacier termini. On an individual level, all seals collected profiles from both the fjord and Kronebreen terminus regions, but only three out of five seals collected profiles close to the terminus of Kongsbreen South. The proportion of profiles collected in the near-terminus regions varied between 4% and 90% for each individual seal, with four of the five seals collecting more than 30% of their dives within these regions.

The near-terminus regions of Kronebreen and Kongsbreen South cover areas of 2.0 km^2^ and 1.6 km^2^ respectively, while the full study area comprises a fjord region of 42.6 km^2^. Given the relative sizes of these areas, only 4–5% of dive profiles would be expected within each of the two near-terminus regions if the seals’ dives were uniformly distributed within the study area. The number of profiles close to Kongsbreen South is close to what would be expected; however, the number of profiles close to Kronebreen is significantly higher, both in total and on an individual basis for four of the five seals. The over-representation of profiles close to the terminus of Kronebreen demonstrates that seals preferentially dived within this region. This supports previous studies which have suggested that calving fronts are favoured by ringed seals^14,15,31^.

The use of GPS-equipped SRDLs allows the location of each dive to be determined more precisely than has been possible in previous studies that depended upon ARGOS location estimates^32^. Figure 2a shows the density of dive profiles close to the terminus of Kronebreen over the full study period. The highest densities of profiles are observed in discrete regions of the terminus. The densest cluster is found in the northern half of the terminus, close to a previously identified location of subglacial discharge^28,29^, and consistent with a plume visible in satellite images from earlier in 2012 (Fig. 2b,c). The smaller clusters to the north and south are also consistent with plume locations identified in recent timelapse imagery^30^.

### Profiles collected within the plume

**Figure 3:**
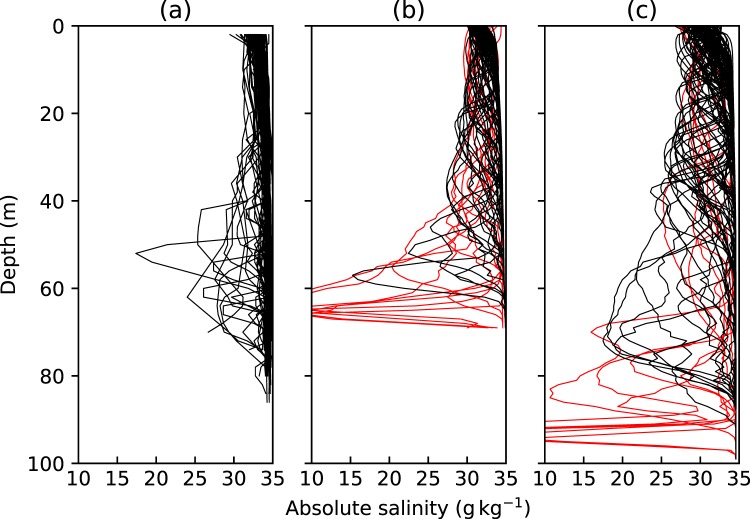
Comparing (**a**) seal-collected salinity to (**b**,**c**) modelled salinity. Salinity data in (**a**) were collected between 1st September and 31st October using GPS-CTD-SRDLs at the terminus of Kronebreen. Randomly sampled modelled salinity data for (**b**) a 70 m deep terminus with 10 m^3^ s^−1^ discharge and (**c**) a 100 m deep terminus with 50 m^3^ s^−1^ discharge. Red lines indicate profiles collected within 10 m of the source of subglacial discharge.

A number of profiles collected within the clusters at the terminus of Kronebreen show spikes of low salinity (Fig. 3a) which are significantly larger than the uncertainty in the data. Similar spikes can also be seen in the temperature, but to a lesser extent (Supplementary Figure S1). These profiles are present in data collected by three out of the five seals. We extracted comparable profiles from a high-resolution plume model at arbitrary distances from the plume source (Fig. 3b,c). These modelled profiles show similar spikes, both in their magnitude and depth. Profiles highlighted in red in Fig. 3b and c were extracted from the model within 10 m of the source of subglacial. Such sharp spikes close to the deepest part of the profile are notably absent from the seal-collected data. Given that the deepest point reached by the seals during their dive is likely to be the depth where they focus their foraging, this suggests that the seals foraged close to, but not within, the plume and only entered the plume itself as they returned to the surface.

**Figure 4:**
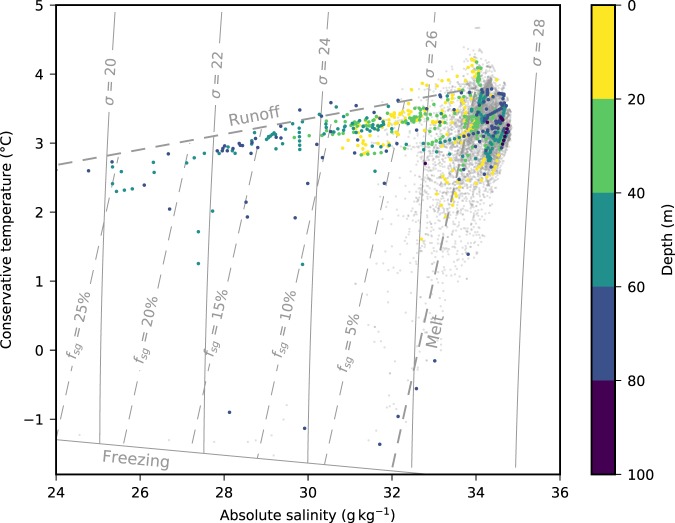
Temperature-salinity (T-S) plot of profiles collected within 500 m of the terminus. Profiles with large spikes in salinity are coloured by depth, while other profiles are shown in grey. Also plotted are the runoff and melting mixing lines, and the estimated fraction of subglacial discharge *f*_*sg*_.

Further evidence that the seals entered the plume can be seen in a temperature-salinity plot of the profiles close to the terminus (Fig. 4). Runoff and meltwater mixing lines as well as the fraction of subglacial discharge were calculated following a previous study^24^. In general, profiles with large spikes in salinity follow the runoff mixing line, showing that the water mass has mixed between the ambient ocean water and freshwater at the pressure melting point. At the deepest points of the dives (80–100 m), properties are generally close to the ambient water. The freshest waters, with the highest fractions of subglacial discharge, are found at depths between 40 and 80 m, with a maximum of 27% at a depth of 64 m. Above these spikes, between 0 and 40 m, the properties move back towards ambient conditions, suggesting continued mixing with ambient ocean water towards the surface. A small number of points with high fractions of subglacial discharge also show a tendency towards the meltwater mixing line, suggesting that these profiles contain a larger volume of glacial meltwater. This is particularly clear in one profile, which appears to follow the meltwater mixing line and the freezing line, before moving back towards the runoff mixing line. Generally, profiles close to the terminus but without spikes in salinity follow the meltwater mixing line as they approach the surface. This is consistent with ambient ocean water mixing with glacial meltwater.

### Temporal variation of dive locations

The temporal variation of the profile clusters around the subglacial outlets at the terminus of Kronebreen was further assessed using the DBSCAN clustering algorithm^33^. For every profile location, the DBSCAN clustering algorithm counts the number of surrounding profiles within a given radius, a point is classified as a cluster if the number of points within this radius exceeds a set threshold value. The two key parameters are therefore the radius and the threshold number of points; a parameter sweep was used to test a range of these parameters and the results are presented here as the percentage of sensitivity tests identifying clustering. The results can therefore be interpreted as the density of clustering, such that 100% shows dense clustering, and 0% shows no clustering. More details on the methods and interpretation can be found in the Methods section.

**Figure 5:**
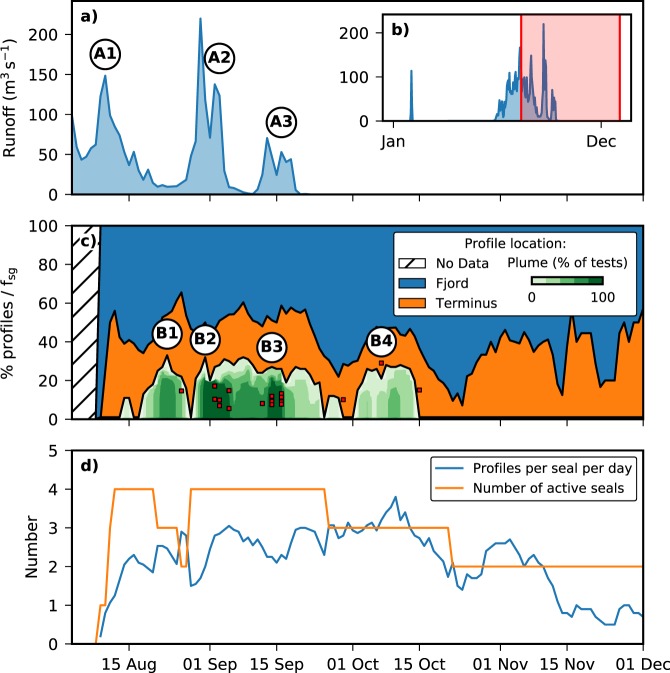
Temporal variation in modelled surface runoff and seal-collected profile locations. (**a**) Runoff simulated using a surface mass balance model integrated over Kronebreen catchment through the study period, inset (**b**) shows the runoff for the full year 2012 with the study period highlighted for context. (**c**) Percentage of CTD profiles collected in the fjord and near-terminus regions and the percentage of sensitivity tests identifying plume clustering at the glacier terminus. Red points indicate profiles which contained spikes of low temperature and salinity and their mean freshwater fraction *f*_*sg*_. (**d**) Number of instrumented seals within the study region and the mean number of profiles collected by each seal per day. Labels A1-3 and B1-4 indicate peaks in the modelled surface runoff and plume clustering respectively.

Supplementary Video S1 shows how the positions of the dives varied through the study period, with profiles identified in clusters highlighted. Figure 5c shows the proportion of profiles in each classification (fjord, terminus or plume) throughout the study period, also highlighted is the timing of profiles collected within the plume and the mean fraction of subglacial discharge in the profile.

The percentage of profiles collected near the terminus varied between 10 and 65% of profiles in all regions. Despite these large fluctuations, no systematic trends were detected through the study period. Though it should be noted that towards the end of the study period there were fewer seals sending data, as well as fewer dives per seal. The proportion of profiles within clusters varied from 0 to 30% of profiles collected. Within this, there were a number of peaks which indicate more or less dense clustering. The second and third peaks, B2 and B3, are identified in 97% and 91% of sensitivity tests respectively, showing the most dense clustering during the study period. Between these two peaks, at least 60% of sensitivity tests continue to identify clustering, suggesting that weaker clustering still occured during this time. The first peak, B1, is identified in a maximum of 72% of the sensitivity tests, suggesting slightly less dense clustering than in B2 and B3. The final peak, B4, also shows a peak containing approximately 30% of the profiles; however, this peak is only identified in a maximum of 57% of the sensitivity tests, indicating that clustering during this time is relatively weak compared to other times during the study period. No clustering was identified in the seal dives after 15th October.

The timing of the profiles with higher subglacial discharge fractions is generally consistent with the peaks identified in the clustering. The two strongest peaks in clustering, B2 and B3, are coincident with the collection of six and eight of the profiles, respectively. Four other profiles were collected during the study period; one shortly followed peak B1, and the other three spread around peak B4. These final three profiles are separated by periods of 5 days, and their consistency with peak B4 is less clear.

Figure 5a shows the modelled surface runoff for Kronebreen catchment during the study period (see Methods). Figure 5b shows the runoff for 2012 in full to set the study period in context. A number of distinct peaks can be identified in the runoff time series. The first peak in the modelled surface runoff (A1 in Fig. 5a) reached 150 m^3^ s^−1^, but this peak occurred during the period in which the seals were being instrumented and therefore cannot be reliably compared to the seal data. The two subsequent peaks in the modelled surface runoff (A2 and A3) of 220 m^3^ s^−1^ and 70 m^3^ s^−1^, respectively, coincided with two peaks in the percentage of sensitivity tests identifying clustering (B2 and B3), as well with the collection of the largest number of profiles within the plume. Between these peaks runoff dropped to between 0 and 10 m^3^ s^−1^. Runoff was at, or close to, 0 m^3^ s^−1^ after 20th September.

## Discussion

### Plume dynamics and glacier hydrology

The temperature and salinity profiles collected by the GPS-CTD-SRDLs show strong evidence for multiple seals having entered the plume on a number of occasions. The consistency of the temperature and salinity spikes with the runoff mixing line in Fig. 4, and the presence of spikes in the data from multiple sensors, rule out instrument error. The density of water in the spikes is lower than the ambient water, meaning that these profiles are unstable and would not typically be found in the open ocean. Additionally, freshwater fractions are generally greater at depth. Both of these observations are consistent with the seals having interacted with a source of buoyant, cold, fresh water released at depth that gradually mixed towards ambient conditions as it rose. Such a source of discharge could only come from the subglacial hydrological system of the glacier. A previous study at Saqqarliup Sermia, a glacier in West Greenland with a terminus around 1.5 times the width and depth of Kronebreen but with a comparable discharge volume, found subglacial discharge fractions between 2 and 10% within 20 m of the surface^24^. The data collected here show subglacial discharge fractions as high as 27% at 64 m below the surface. This is comparable to the data collected in Greenland, but demonstrates that ringed seals are able to access the upwelling core of the plume at a depth where, to date, data has not been collected by traditional CTDs^23,24^.

It is difficult to make useful inferences about the discharge volume and melt rate from each individual profile because the data are instantaneous profiles from a turbulent and rapidly fluctuating plume. However, the clustering analysis provides a valuable insight into the intra-annual variation of the plume at Kronebreen when compared with modelled surface runoff from the catchment. The two peaks in modelled surface runoff, A2 and A3, coincide closely with peaks B2 and B3 in the seal clustering analysis and with the increase in profiles which contain high freshwater fractions. This is unlikely to be coincidental, and suggests a rapid linkage between increases in surface runoff on the glacier, its release at the terminus, and the response of the seals to a time-varying food supply. A short delay such as this in the glacial hydrological system is indicative of well-developed surface-to-bed connectivity and an efficient subglacial drainage system. This is consistent with the dense clustering of seals at the terminus in B2 and B3, which suggests that the plume is forced by a point source of discharge from a channelised subglacial drainage system at the terminus. The data in our study were collected from August onwards, therefore these results are in agreement with previous studies of Alpine and Greenlandic glaciers, which suggest that a channelised subglacial system would have developed by late summer^34–36^.

There were also periods when the seals continued to dive in less dense clusters in the same locations despite modelled surface runoff being low. Peaks B1 and B4 and the sustained levels of clustering between B2 and B3 are examples of this situation. This could be attributed to a delay in the response of the seals to the plume ceasing; however, spikes in the temperature and salinity data at depth are still present in the dive profiles and, additionally, the spatial and temporal variation of the seals’ dive locations (Supplementary Video S1) suggests that the seals respond quickly to changes in the position of the plumes. Therefore, this is more likely to represent behaviour in the hydrological system, which could result from some combination of two factors: (1) retention of runoff on the glacier surface, and (2) modulation of runoff from the higher areas of the catchment within the subglacial drainage system. Recent work has shown evidence for storage and modulation of water in the subglacial hydrological system at Kronebreen^30^.

### Seal behaviour

Our results show that ringed seals in Kongsfjorden strongly favour the terminus of Kronebreen, a marine-terminating glacier, as a foraging area over the nearby Kongsbreen South, a primarily land-terminating glacier. Within the terminus region the seal dive profiles were clustered in specific locations. The locations of the clusters are consistent with previously observed locations of subglacial discharge^28–30^ and with plume locations visible in coincident satellite imagery (Fig. 2). Further evidence for the seals entering the plume is found in the salinity profiles collected by the GPS-CTD-SRDLs, which show subglacial discharge fractions as high as 27% at depths greater than 40 m.

The seals’ preference for foraging close to the plumes is likely driven by an abundant supply of food in, or very close to, these features. The presence of abundant food linked to plumes is in agreement with other work that has also observed high concentrations of seabirds feeding at the surface of plumes^13^. While this dataset cannot definitively identify the food source that attracts ringed seals, a number of insights into the behaviour of the seals can be gained. The seals appear to forage in a slightly larger radius around the plume than the seabirds at the surface. In most of the seal-collected profiles, the temperature and salinity are close to ambient conditions at the lowest point of the dive. This suggests that the seals forage near the boundary of the plume, which is in contrast to the seabirds at the surface that focus their attention close to the centre of the plume.

Spikes of low temperature and salinity were observed in the water column during the seals’ ascent towards the surface. This suggests either (i) the seals enter the plume and quickly leave it again due to the high turbidity, producing the large spikes in the data, or (ii) the seals return to the surface vertically and pass through turbulent eddies of fresher water within the plume as they ascend. Vertical profiles extracted from a high-resolution plume model produced patterns in temperature and salinity that were very similar to those seen in the seal data, suggesting that (ii) is the most likely explanation. The profiles extracted from the model results also demonstrate that the seals must be within 10–20 m of the source of subglacial discharge in order to produce spikes in temperature and salinity of such magnitude at that depth, but also that in general the seals terminate their dives outside the plume and only enter the plume while they are returning to the surface.

It is clear from the temporal variation of dive locations that the seals respond rapidly to changes in the volume and location of subglacial discharges. The temporal coincidence of peaks A2 and A3 in the modelled surface runoff with peaks B2 and B3 in the seal clustering, in combination with the changes in the location of clustering in Supplementary Video S1, show that the seals respond to changes in the position and strength of the plume within hours to days. This implies that prey for the seals is available quickly following the onset of a plume, rather than taking time to establish or move from other locations. The peaks in clustering also suggest that the availability of prey in or close to the plume is linked to the volume of subglacial discharge released from the glacier.

## Conclusions

The results of our study provide the first dataset collected at depth within the upwelling region of a plume of subglacial discharge; the oceanographic data collected in and around the plume span a four-month period between August and December 2012. The data show evidence for a channelised and efficient subglacial system towards the end of summer at Kronebreen. At the same time, the hydrological system of the glacier still has capacity for the delay and storage of water, which is subsequently released during periods of low surface runoff. We have shown that the foraging behaviour of ringed seals at Kronebreen is focussed at, and significantly affected by, the release of subglacial discharge from the glacier terminus. Ringed seals preferentially foraged in an area within a few hundred meters of the plume, and, on a number of occasions during our study period, entered the plume itself. This behaviour suggests that plumes, and not just the glacier front, exert an important influence on the ecosystem within Kongsfjorden.

The continued retreat of Kronebreen, as well as other tidewater glaciers in Svalbard and more generally, will eventually lead to the loss of marine-termini as the glaciers retreat onto land. This change will result in the disappearance of an important foraging area for ringed seals and other marine species, as well as affecting fjord circulation, nutrient budgets and plankton availability. Our results demonstrate the value of instrumenting ringed seals with GPS-CTD-SRDLs, both for monitoring the response of tidewater glaciers to future changes in climate and for understanding the seals’ responses to these changes in their habitat.

## Methods

### Seal-collected oceanographic data

Oceanographic data were collected using GPS-CTD-SRDLs^26^. These instruments were glued to the fur of ringed seals in Kongsfjorden, Svalbard, (Fig. 1) during August 2012^31^. Instrumenting of the seals was carried out in strict accordance with the recommendations of the Norwegian Animal Care Authority (Forsøksdyrutvalget) and was approved under permit number 2010/45416. The protocol was also approved by the Governor of Svalbard (Sysselmannen på Svalbard) under permit number 2011/01095-69 a.512 and followed best practice for all animal handling.

The GPS-CTD-SRDLs were configured to collect temperature, practical salinity and depth (pressure) on the ascending portion of the dive, and to transmit data from the deepest dive within each four-hour period via the Argos satellite network. Data were converted to conservative temperature (Θ) and absolute salinity (*S*_*A*_) to be consistent with TEOS-10 standard^37^. The devices deployed in 2012 used the Fastloc-GPS system, in addition to position estimates from the Argos network, to improve the accuracy of geolocation data. Dive profile locations were interpolated from GPS fixes acquired before and after dives using the timing between the dive and the bracketing GPS fixes^38,39^. This can lead to low positional accuracy if the seal moves over large distances between dives, but when the seals are repeatedly diving in the same location, for example when dive profiles are clustered around the plume, the positional accuracy of the dive profiles is similar to that from the GPS device.

The data were filtered to remove erroneous or inaccurate data using standard Argo float processing procedures^40^. Additionally, the data were filtered to remove profiles where more than three consecutive locations had been interpolated from widely spaced GPS fixes, and profiles where temperature and salinity data had been interpolated within the profile. After filtering, a total of 648 profiles were obtained within the study area between 1st August and 31st December 2012.

The temperature and salinity data from the seal dive profiles have a number of sources of uncertainty. Under laboratory conditions, the instruments collect data with an accuracy of ±0.005 °C and ±0.02 g kg^−1^ for temperature and salinity respectively^26^. However, the deployed sensors are also subject to uncertainties due to pressure effects and an external field effect on the conductivity sensor, which cannot be corrected for in advance. In previous studies, these effects have been shown to reduce the accuracy of the sensors to ±0.02 °C for temperature and ±0.1 g kg^−1^ for salinity^41^, while another study found offsets of −0.3 g kg^−1^ in the reported salinity values^26^. Delayed-mode quality control procedures exist to address these uncertainties^41^; however, the analysis in this paper considers variations in the temperature and salinity data which are one to two orders of magnitude larger than these uncertainties. Therefore, further delayed-mode quality control procedures to correct for these effects were not applied to the data presented here and the uncertainties should be expected to be similar to previous studies^26,41^.

### Clustering analysis of dive locations

Clustering of profiles was identified using the DBSCAN algorithm^33^, a density-based clustering algorithm. The DBSCAN algorithm uses two parameters: *eps*, the radial distance to search around each point; and *minPts*, the minimum number of points which must lie within the radius for the central point to be classified as a cluster point. The identification of a plume cluster is sensitive to the selection of these two parameters. To account for this sensitivity, a Monte Carlo style parameter sweep was used to test a range of values for *eps* and *minPts*. We selected values of *eps* in the range 25 to 200 m, and *minPts* in the range 4 to 10. Parameter combinations were selected randomly from a uniform distribution within the ranges specified and tests were run for 1000 different parameter combinations. The temporal variation of plume clustering was derived using a 5-day moving window on the seal profile locations. The window was stepped by one day with the DBSCAN algorithm run at each time step.

The presence of a plume cluster is presented here as a percentage of the number of random parameter combinations tested in the parameter sweeps. Therefore, 100% indicates that plume clusters were identified in all parameter combinations tested and 0% indicates that no plume clusters were identified with any parameter combinations. The physical interpretation of this percentage value relates to the parameter ranges tested. As such, if the plume is identified in a low percentage of tests these will be tests with low values of *eps* and high values of *minPts*; for example, the lowest possible threshold for a cluster with these parameter ranges is 4 profiles within a 200 m radius. This is a low threshold and, as such, would represent very weak clustering which may not necessarily be associated with a plume. At the other end of the spectrum, the highest threshold for a cluster is 10 profiles within a 25 m radius. To identify clustering in a high percentage of sensitivity tests, the results need to be approaching this threshold; therefore, a high percentage of sensitivity tests suggests that the profiles are very densely clustered, which is very likely indicative of the presence of a plume.

### Runoff data

A surface energy balance model coupled with a subsurface snow model^42,43^ was adapted to simulate surface runoff from the entire Kongsfjorden basin. An area mask was used to extract surface runoff from Kronebreen catchment which drives the plume at the terminus. The surface energy balance model was calibrated with automatic weather station data and observational winter and summer mass balance data from surface ablation stakes. Precipitation forcing was extracted from ERA-interim data which was downscaled to 1 km resolution and then calibrated using winter mass balance data for Svalbard^44^. Other climatic parameters came from a nearby meteorological site at Ny-Ålesund.

We assume that modelled surface runoff can be used as a proxy for discharge at the terminus^21,45^. This assumption makes no allowance for any flow delays or retention; either on the surface or subglacially. This assumption is reasonable close to the terminus, where it is likely that water could access a well-developed subglacial system rapidly, particularly in the late summer season considered here. This would thus allow runoff to reach the terminus within the one-day time step used to analyse the seal data. However, further from the terminus the flow delay in the hydrological system is likely to be larger. As a result, the true discharge at the terminus is likely to have slightly lower peaks and reduced gradients in the falling limbs as flow from the higher regions of the catchment is modulated in the hydrological system.

### Modelled plume behaviour

We ran a high-resolution plume model using the fluid dynamics code Fluidity^46^ to provide a comparison to seal-collected CTD profiles collected within the plume. The model setup is described in more detail in Supplementary Text S2. Two terminus depths of 70 m and 100 m were used to reflect the range of water depths at the terminus of Kronebreen. The model was initialized using a spatial and temporal average of temperature and salinity data collected by the GPS-CTD-SRDLs in the main fjord before 20th September. It is assumed that this is representative of the ambient ocean water into which the plume was released.

A number of representative vertical profiles were extracted from randomly selected points in time and space within the model to simulate the seal-collected CTD profiles. The discharge volume at the time of each seal dive is not known, and the turbulent fluctuations mean that the properties at any fixed point vary rapidly. For these reasons, the model results are used for qualitative comparisons to the seal data and are not expected to reproduce the profiles collected by the GPS-CTD-SRDLs exactly.

## Electronic supplementary material

Supplementary Information

Supplementary Video

## Data Availability

Seal-collected oceanographic data analysed in this study are available from the Norwegian Polar Institute via the Norwegian Polar Data Centre (10.21334/npolar.2017.7b538020). The fluid dynamics code Fluidity is freely available from http://fluidityproject.github.io/. The model setup files, meshes and other data used in this study are available from the authors upon request.
