# Supplementary material for: Subglacial discharge plume behaviour revealed by CTD-instrumented ringed seals
Source: Sci Rep. 2018 Sep 7;8:13467. doi: 10.1038/s41598-018-31875-8 (PMC6128829; doi:10.1038/s41598-018-31875-8)
Supplement: Supplementary file 1 — Supplementary Information [file 41598_2018_31875_MOESM1_ESM.pdf]

Supplementary Information for "Subglacial discharge plume behaviour revealed by CTD-instrumented ringed seals"

Alistair Everett[1,*], Jack Kohler[1], Arild Sundfjord[1], Kit M. Kovacs[1], Tomas Torsvik[1], Ankit Pramanik[1,2], Lars Boehme[3], and Christian Lydersen[1]

[1]Norwegian Polar Institute, Fram Centre, N-9296 Tromsø, Norway
[2]ESSO-National Centre for Antarctic and Ocean Research, Headland Sada, Vasco da Gama, Goa- 403804, India
[3]NERC Sea Mammal Research Unit, Scottish Oceans Institute, University of St Andrews, UK
[*]Contact author: alistair.everett@npolar.no

Supplementary text

Text S1: Estimating plume freshwater fraction

The freshwater fractions in the plume were estimated using a three-water-mass mixing model, following a previous study[1]. We assume that the subglacial discharge and meltwater are both fresh (i.e. $S_{Asg} = S_{Amw} = 0\,\mathrm{g\,kg^{-1}}$), the temperature of the subglacial discharge Θ_{sg} is at the pressure melting point -0.07°C, and the ice temperature Θ_i is -15°C. To calculate the slope of the meltwater mixing line, a virtual temperature Θ_{mw} must be calculated taking into account the energy required to melt the ice. This is calculated using

$$\Theta_{mw} = \Theta_{sg} - \frac{L}{c_w} - \frac{c_i}{c_w}(\Theta_{sg} - \Theta_i), \quad (1)$$

where L is the latent heat of fusion (334 kJ kg^{-1}), c_w is the heat capacity of seawater (3947 J kg^{-1}) and c_i is the heat capacity of ice (2009 J kg^{-1}). The following equations can then be derived and used to estimate the meltwater fraction f_{mw} and the subglacial discharge fraction f_{sg}:

$$f_{mw} = 1 - f_{sg} - \frac{S_A}{S_{Ae}}, \quad (2)$$

and

$$f_{sg} = \frac{1}{\Theta_{sg} - \Theta_{mw}} \left(\Theta - \Theta_{mw} \left[1 - \frac{S_A}{S_{Ae}} \right] - \Theta_e \frac{S_A}{S_{Ae}} \right), \quad (3)$$

where the variables and their chosen values are described below.

To illustrate subglacial discharge volumes at depth, we took conservative temperature and absolute salinity values of $\Theta = 2.5$°C and $S_A = 24.8\,\mathrm{g\,kg^{-1}}$ from the spike which occurs at 60 metres depth. The temperature and salinity of the ambient waters entrained into the plume were determined from the average properties of profiles without spikes at the same depth. This gave properties of $\Theta_e = 3.8$°C and $S_{Ae} = 34\,\mathrm{g\,kg^{-1}}$. Using these values in Equation 1 and 2 gives a subglacial discharge fraction of 27 % and a meltwater fraction of 0.3 %.

Text S2: High-resolution plume model

The high-resolution plume model used here is based upon the fluid dynamics code Fluidity[2] which solves the Navier-Stokes equations on a fully unstructured three-dimensional finite element mesh. The model formulation builds upon previous work[3], with the addition of a large eddy simulation (LES) turbulence model[4] and the use of the synthetic eddy method (SEM)[5] at the inlet.

The model domain is semicylindrical with a radius of 200 m, where the flat face represents the ice front and the cylindrical face represents the boundary with the fjord. Two ice front depths of 70 and 100 m were tested. The mesh resolution is 1 m at the centre of the cylinder and decreases radially to 5 m at the fjord boundaries. Close to the inlet, the mesh is further refined to a resolution of 0.5 m.

Dirichlet boundaries are used for the top and bottom surfaces. On the top surface, a no-normal flow, free-stress condition is prescribed and the hydrostatic reference pressure is set to zero. On the bottom and ice front surfaces a no-slip boundary

condition is prescribed by setting all components of the velocity to zero. The ocean boundaries are stress free, where the normal component of shear stress across the boundary is set to zero.

A horizontally facing inlet at the foot of the ice boundary is used to release subglacial discharge into the domain. The channel is assumed to be semi-circular and we use the relationship

$$X = \left(\frac{c_1}{c_2 \, c_3^2 \, N_{\mathrm{eff}}^n} \right)^{2/7} Q^{6/7}, \tag{4}$$

to determine the cross-sectional area X of the outlet from the discharge Q and the effective basal pressure N_{eff}, where c_1, c_2, c_3 and n are constants[6,7]. To produce fully turbulent conditions at the inlet we use the synthetic eddy method (SEM)[5]. When used with an LES turbulence model, an SEM inlet produces a more rapid transition to turbulent flow, and therefore more realistic plume behaviour, than a uniform velocity at the inlet[8].

A three-equation model is implemented in Fluidity to calculate melt rates on the ice-front[9,10]. We also apply a background velocity of $10^{-4}\,\mathrm{m\,s^{-1}}$ to simulate melt in the absence of convection. The model setup files and meshes used in this study are available from the authors upon request.

Supplementary figures

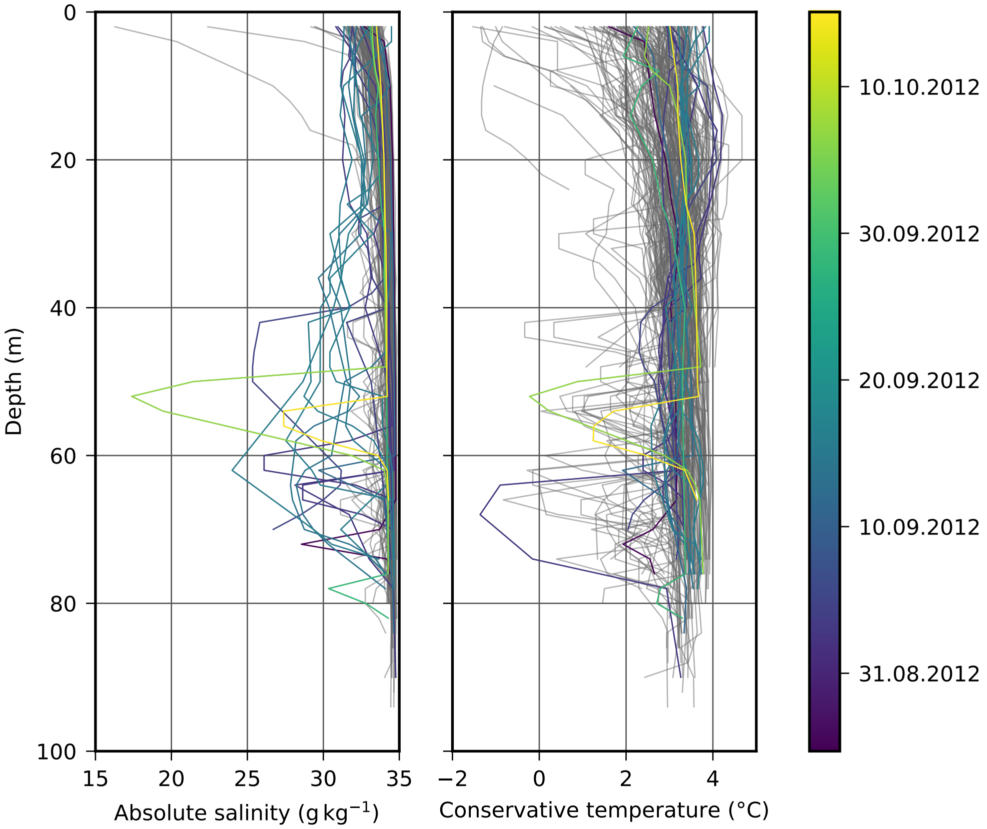

Figure S1. Absolute salinity and conservative temperatures collected by ringed seals within 500 metres of the terminus of Kronebreen. Profiles with high freshwater discharge fractions have been highlighted and coloured by the date they were collected. Spikes in temperature which are not associated with low salinity are due to high meltwater fractions, rather than subglacial discharge.

Supplementary tables

Table S1. Ringed seals instrumented in this study including the first and last transmission dates and the number of CTD profiles collected in the study area between 14th August and 31st December 2012.

Seal ID	Date tagged	Last transmission	Profiles
F61-Dot-12	14.08.2012	21.10.2012	212
F61-Grete-12	16.08.2012	29.04.2013	23
F64-Garbo-12	17.08.2012	10.02.2013	193
M10-Samson-12	24.08.2012	22.02.2013	62
M60-Derek-12	14.08.2012	27.04.2013	180
	Total number of profiles collected:		670

References

1. Mankoff, K. D. *et al.* Structure and dynamics of a subglacial discharge plume in a Greenlandic fjord. *J. Geophys. Res. Ocean.* **121**, 8670–8688 (2016). DOI 10.1002/2016jc011764.
2. Piggott, M. D. *et al.* A new computational framework for multi-scale ocean modelling based on adapting unstructured meshes. *Int. J. Numer. Methods Fluids* **56**, 1003–1015 (2008). URL http://dx.doi.org/10.1002/fld.1663. DOI 10.1002/fld.1663.
3. Kimura, S., Candy, A. S., Holland, P. R., Piggott, M. D. & Jenkins, A. Adaptation of an unstructured-mesh, finite-element ocean model to the simulation of ocean circulation beneath ice shelves. *Ocean. Model.* **67**, 39–51 (2013). URL http://dx.doi.org/10.1016/j.ocemod.2013.03.004. DOI 10.1016/j.ocemod.2013.03.004.
4. Smagorinsky, J. General Circulation Experiments With The Primitive Equations. *Mon. Weather. Rev.* **91**, 99–164 (1963). URL http://dx.doi.org/10.1175/1520-0493(1963)091<0099:GCEWTP>2.3.CO;2. DOI 10.1175/1520-0493(1963)091<0099:gcewtp>2.3.co;2.
5. Jarrin, N., Benhamadouche, S., Laurence, D. & Prosser, R. A synthetic-eddy-method for generating inflow conditions for large-eddy simulations. *Int. J. Heat Fluid Flow* **27**, 585–593 (2006). URL http://dx.doi.org/10.1016/j.ijheatfluidflow.2006.02.006. DOI 10.1016/j.ijheatfluidflow.2006.02.006.
6. Slater, D. A., Nienow, P. W., Cowton, T. R., Goldberg, D. N. & Sole, A. J. Effect of near-terminus subglacial hydrology on tidewater glacier submarine melt rates. *Geophys. Res. Lett.* **42** (2015). URL http://dx.doi.org/10.1002/2014GL062494. DOI 10.1002/2014gl062494.
7. Schoof, C. Ice-sheet acceleration driven by melt supply variability. *Nat.* **468**, 803–806 (2010). URL http://dx.doi.org/10.1038/nature09618. DOI 10.1038/nature09618.
8. Keating, A., Piomelli, U., Balaras, E. & Kaltenbach, H.-J. A priori and a posteriori tests of inflow conditions for large-eddy simulation. *Phys. Fluids* **16**, 4696 (2004). URL http://dx.doi.org/10.1063/1.1811672. DOI 10.1063/1.1811672.
9. Holland, D. M. & Jenkins, A. Modeling Thermodynamic Ice-Ocean Interactions at the Base of an Ice Shelf. *J. Phys. Oceanogr.* **29**, 1787–1800 (1999).
10. Kimura, S., Holland, P. R., Jenkins, A. & Piggott, M. The effect of meltwater plumes on the melting of a vertical glacier face. *J. Phys. Ocean.* **44**, 3099–3177 (2014). URL http://dx.doi.org/10.1175/JPO-D-13-0219.1. DOI 10.1175/jpo-d-13-0219.1.
